# A Novel β-Glucosidase From *Chryseobacterium scophthalmum* 1433 for Efficient Rubusoside Production From Stevioside

**DOI:** 10.3389/fmicb.2021.744914

**Published:** 2021-10-12

**Authors:** Zhenxin Yan, Xueting Cao, Xiao Yang, Shida Yang, Li Xu, Xukai Jiang, Min Xiao

**Affiliations:** ^1^State Key Laboratory of Microbial Technology, Shandong University, Qingdao, China; ^2^National Glycoengineering Research Center, Shandong Key Laboratory of Carbohydrate Chemistry and Glycobiology, NMPA Key Laboratory for Quality Research and Evaluation of Carbohydrate-Based Medicine, Shandong University, Qingdao, China

**Keywords:** β-glucosidase, rubusoside, stevioside, crude steviol glycosides extract, *Chryseobacterium scophthalmum*

## Abstract

As a natural sweetening and solubilizing agent, rubusoside has great potential in the application of healthy beverages and pharmaceuticals. However, the direct extraction and purification of rubusoside from raw materials is inefficient. In this work, a novel β-glucosidase (*Cs*BGL) was obtained from *Chryseobacterium scophthalmum* 1433 through screening of the environmental microorganisms. *Cs*BGL markedly hydrolyzed sophorese (Glcβ1-2Glc) and laminaribiose (Glcβ1-3Glc), but for steviol glycosides, it only hydrolyzed the C-13/C-19-linked sophorese, instead of the C-13/C-19-linked Glcβ1-2[Glcβ1-3]Glc trisaccharide and Glcβ1-monosaccharide. It efficiently hydrolyzed stevioside (240 g/L) to produce rubusoside (99% yield) at 47.5°C for 70 min. Even when using a crude steviol glycosides extract (500 g/L) containing ∼226 g/L stevioside as the substrate, *Cs*BGL could also convert stevioside to rubusoside (99% yield) at 47.5°C for 2 h, in which the rubusoside concentration increased from the initial 42 g/L to the final 222 g/L. These results reveal that *Cs*BGL would be a promising biocatalyst for the industry-scale production of rubusoside from stevioside or/and the crude steviol glycosides extract.

## Graphical Abstract

As a natural sweetening and solubilizing agent, rubusoside has great potential in the application of healthy beverages and pharmaceuticals. A novel β-glucosidase CsBGL obtained here from *Chryseobacterium scophthalmum* 1433 through screening of the environmental microorganisms could specifically hydrolyze the C-13- or/and C-19-linked sophorese of steviol glycosides, but not the C-13- or/and C-19-linked Glcβ1-2[Glcβ1-3]Glc trisaccharide and Glcβ1-monosaccharide of steviol glycosides, and thus could be a promising biocatalyst for efficient rubusoside production from stevioside.

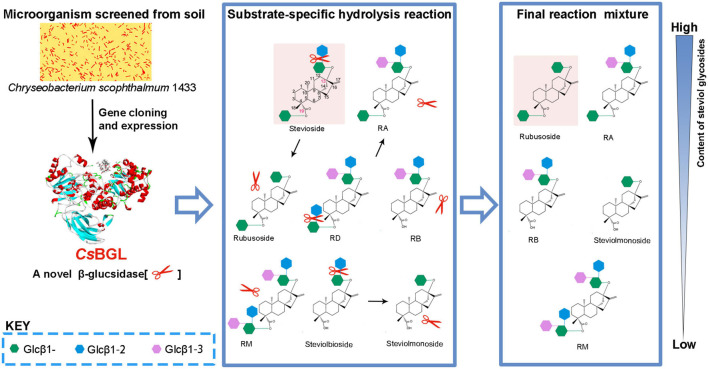

## Introduction

Rubusoside is a natural non-caloric sweetener and mainly extracted from *Rubus suavissimus* S. Lee which only grows in the southern area of China (Ko et al., 2012). It is approximately 114 times sweeter than sucrose (Darise et al., 1984) and displays diverse bioactivities, such as hypoglycemic, antiallergic, anticariogenic, and antiangiogenic functions (Liu et al., 2006, 2020; Kim et al., 2019; Wang et al., 2020). Therefore, rubusoside has been widely used as an additive to herbal tea and healthy beverages. On the other hand, the amphipathicity of rubusoside makes it a natural solubilizer to enhance the solubility of various pharmaceutical compounds, including paclitaxel, resveratrol, curcumin, capsaicin, cyclosporine, nystatin, etoposide, and erythromycin (Ko et al., 2012; Zhang et al., 2012, 2017). However, the direct extraction and purification of rubusoside from the dried leaves of *R. suavissimus* S. Lee is laborious and time-consuming due to its fractional content in raw materials (∼5%) (Chou et al., 2009; Wang et al., 2015). Therefore, the large-scale industrial production of rubusoside remains challenging.

Rubusoside is a minor component of steviol glycosides which belong to tetracyclic diterpene glycosides extracted from the leaves of the *Stevia rebaudiana* Bertoni. The US Food and Drug Administration and the European Food Safety Authority have identified steviol glycosides as “generally recognized as safe” (GRAS) compounds (Gerwig et al., 2016). Stevioside is the major component of steviol glycosides and has been manufactured commercially since the 1970s (Ko et al., 2012). Stevioside and rubusoside share the same skeleton; the only difference is that stevioside has an additional β-D-glucopyranosyl unit in a β-1,2-linkage at the C-13 hydroxyl group. Therefore, it is feasible to convert stevioside into rubusoside by selectively cleaving the extra glucopyranosyl unit at the C-13 hydroxyl group of stevioside (Ko et al., 2012; Wan et al., 2012; Wang et al., 2015; Lan et al., 2019).

Several β-glucosidases and β-galactosidases were found to be able to hydrolyze the β-1,2-glucosidic bond in the sophorosyl disaccharide at the C-13 hydroxyl group of stevioside (Ko et al., 2012; Wan et al., 2012; Nguyen et al., 2014; Boonkaew et al., 2019). Unfortunately, most of these enzymes, for example, SSGase from Sigma-Aldrich’s commercial enzyme cocktails (Ko et al., 2012) β-galactosidase from *Aspergillus* sp. CICIMF0620 (Wan et al., 2012) and lactase from *Thermus thermophilus* (Nguyen et al., 2014) display promiscuous hydrolysis activity to stevioside and other steviol glycosides, including rubusoside. This inevitably decreases the yield of rubusoside because the rubusoside produced is further hydrolyzed. Although past studies claimed that BGL1 from *Streptomyces* sp. GXT6 and SPBGL1 from *Sphingomonas elodea* ATCC 3146 specifically hydrolyze stevioside to rubusoside and have no hydrolysis activity to rubusoside (Wang et al., 2015; Lan et al., 2019) the reactions catalyzed by these two enzymes took a long time of 6 h to achieve the highest yield of rubusoside. To meet the increasing demand for rubusoside, it becomes essential to find novel enzymes with better substrate specificity and catalytic properties.

The present study aims to discover novel enzymes and explore the optimal catalysis-based reaction system for production of rubusoside from stevioside. Through screening the soil microorganisms, we isolated a new strain of *Chryseobacterium scophthalmum* 1433 which was able to produce a novel β-glucosidase (named *Cs*BGL). *Cs*BGL showed strict hydrolytic specificity to the β-1,2-glucosidic bond in the sophorosyl disaccharide without β-1,3-glucosidic bond branch and did not hydrolyze the single β-D-glucopyranosyl unit in steviol glycosides. The enzyme could efficiently produce rubusoside using stevioside and/or the crude steviol glycosides extract as the substrates, with 99% conversion of stevioside and 99% yield of rubusoside, providing a great potential as an efficient catalyst for the industrial-scale production of rubusoside.

## Materials and Methods

### Materials and Enzymes

Rebaudioside D (RD), Rebaudioside M (RM), Rebaudioside A (RA), stevioside, and the crude steviol glycosides extract containing about 45% stevioside were gifts from Zhucheng Haotian Pharm Co., Ltd. (Weifang, China). Rubusoside and steviol were purchased from Chengdu Herbpurify Co., Ltd. (Chengdu, China). Steviolmonoside, steviolbioside, and Rebaudioside B (RB) were prepared by alkaline hydrolysis from rubusoside, stevioside, and RA, respectively, with 0.5 N NaOH at 100°C for 15 min in this work (Nakano et al., 1998). *p*-Nitrophenyl-β-D-glucopyranoside (*p*NP-β-Glc), *p*-nitrophenyl-N-acetyl-β-D-glucosamine (*p*NP-β-Glc NAc), *p*-nitrophenyl-β-D-glucuronic acid (*p*NP-β-GlcA), *p*-nitrophenyl-β-D-galactopyranoside (*p*NP-β-Gal), *p*-nitro -phenyl-β-fucose (*p*NP-β-Fuc), *p*-nitrophenyl-β-manno -pyranoside (*p*NP-β-Man), *p*-nitrophenyl-α-D-glucopyranoside (*p*NP-α-Glc), and *p*-nitrophenyl-α-D-galactopyranoside (*p*NP-α-Gal) were purchased from Sigma-Aldrich (United States). Esculin, cellobiose, laminaribiose, and gentiobiose were purchased from J&K Scientific Co., Ltd. (Beijing, China). The Phusion DNA polymerase was KOD-Plus-Neo from Toyobo Co., Ltd., Life Science Department (Osaka, Japan). T5 Exonuclease and restriction enzymes were from New England Bio-Labs. Glucose Assay Kit was from Biosino Bio-Technology and Science Inc. (Beijing, China). The other chemicals used were analytical grade.

### Strains and Media

*Chryseobacterium scophthalmum* 1433 was isolated from a soil sample in a wood. The selective medium was the modified M9 medium, containing 0.5 g NH_4_Cl, 3.0 g Na_2_HPO_4_, 1.5 g KH_2_PO_4_, 1 mM MgSO_4_, and 15 g stevioside in 1 L ultrapure water (pH 7.4). *C. scophthalmum* 1433 has been deposited in the China General Microbiological Culture Collection Center (CGMCC) with an accession number CGMCC 20188. *Escherichia coli* strain BL21(DE3) harboring plasmids were cultured at 37°C in LB medium with 50 μg/ml kanamycin.

### Isolation and Identification of Microorganism

The collected soil sample (1 g) was suspended in 10 ml sterile water, and the suspension was serially diluted and spread on selective media plates. After incubating at 30°C for 7 days, 48 colonies with distinct morphological features were isolated and cultured in liquid selective media at 30°C with shaking at 200 rpm for 7 days. The cultured supernatant was analyzed by thin layer chromatography (TLC) for the hydrolyzed product of stevioside. A colony with the hydrolysis activity to stevioside was selected and its pure culture (strain 1433) was obtained by the streak plate method.

The draft genome of strain 1433 was sequenced on the Illumina HiSeq 4000 platform performed by Sangon Biotech Co., Ltd. (Shanghai, China). Raw reads were generated through base calling, which transforms pyroluminescence intensity signals into nucleotide sequences. High-quality clean reads were obtained by stringently filter raw reads using SOAPnuke software.^1^ The phred quality score should be more than 30 (base call accuracy more than 99.9%). Clean reads were assembled by SPAdes (Bankevich et al., 2012) and the gaps of contigs were repaired by GapFiller (Boetzer and Pirovano, 2012). The proteome of strain 1433 was predicted based on its draft genome. The sequence has been deposited in GenBank with an accession number JAFLHF000000000.1. The draft-genome phylogenomic tree was constructed with the CVTree 3.0 server using the parameter-free and whole-genome-based compositional vector algorithm (Zuo and Hao, 2015). *Elizabethkingia miricola* was used as the outgroup in the phylogenetic analysis. The full-length 16S rRNA gene sequence of strain 1433 (GenBank no. MW689256.1) was generated from the draft genome. Sequence identity analysis was performed using BLAST.^2^

### Peptide Mapping and Sequence Analysis of β-Glucosidase

Cells of *C. scophthalmum* 1433 were harvested from the selection medium by centrifugation. The culture solution was concentrated and washed by ultrafiltration. The cells were lysed through sonication, and the lysate was centrifuged at 15,000 × *g* for 15 min at 4°C. The culture solution, cells, and soluble-lysate were used for stevioside hydrolysis test at 37°C for 10 min in 50 mM sodium phosphate buffer (pH 7.4). The soluble-lysate was used as the crude enzymes in native acidic polyacrylamide gel electrophoresis (native acidic PAGE) (Pompeu et al., 2015). The native electrophoresis under acidic conditions was performed using 3.1% stacking gel and 7.7% running gel. The electrophoretic run (Mini-PROTEAN, Bio-Rad, Hercules, CA, United States) was performed at 4°C. A pre-race was performed under a constant current of 3.0 mA for 30 min, and then samples were loaded and the electrodes were reversed under constant voltage of 120 V for 3 h. Proteins in the gel were visualized by Coomassie brilliant blue (CBB) R-250 staining. The β-glucosidase activity in the gel was detected by staining with a chromogenic solution containing 0.5% (w/v) esculoside and 0.05% (w/v) ferric ammonium citrate at 40°C for 15 min (Edberg et al., 1976). The brown band in the gel was excised and cut to ∼1 mm^3^ slices and decolorized by sequentially washing with ultrapure water, 100 mM NH_4_HCO_3_, 50% acetonitrile, and 100% acetonitrile. Subsequently, the gel slices were reduced by 20 mM dithiothreitol at 55°C for 1 h, alkylated by 100 mM iodoacetamide, avoiding light at 25°C for 30 min, digested by 10 ng/μl trypsin at 37°C for 12 h, extracted by ultrasonic method for 1 h, and desalted by C18 ZipTip pipette tips (Jin-ao et al., 2017). Then the digested peptides were analyzed by prominent nano LC-LTQ orbitrap velos pro ETD; the mass spectral data were searched against the predictive proteome of *C. scophthalmum* 1433 by the Proteome Discoverer Software Work Station 2.3.

The amino acid sequences of *Cs*BGL were used to perform the multiple sequence alignment with three reported β-glucosidases that have hydrolysis activity to stevioside, including BGL1 from *Streptomyces* sp. *GXT6* CGMCC 7091 (GenBank no. KJ958924), (Wang et al., 2015) SPBGL1 from *S. elodea* ATCC 31461 (GenBank no. WP_029622673.1), (Lan et al., 2019) and EcBgl from *Enterococcus casseliflavus* strain EC20 (GenBank no. ECBG_01478), (Boonkaew et al., 2019) and three well studied GH3 β-glucosidases including JMB19063 from a compost metagenome (PDB code: 3U48), (McAndrew et al., 2013) *Bt*BGL from *Bacteroides thetaiotaomicron* (PDB code: 5xxn), (Ishiguro et al., 2017) and *Li*BGL from *Listeria innocua* (GenBank no. CAC97071.1) (Nakajima et al., 2016). Clustal X was used to conduct multiple sequence alignment.^3^

Homology modeling of *Cs*BGL was performed using SWISS-MODEL^4^, and the 3D structure of *Bt*BGL (PDB code: 5xxn) from *B. thetaiotaomicron* served as the template (Ishiguro et al., 2017). *Cs*BGL shared 60.9% sequence identity with the template. The docking studies of the interaction between the *Cs*BGL and stevioside were carried out using Autodock4.2.6.^5^ The docking results were generated and the best conformational pose was scrutinized for analysis by PyMol 1.3. The interactions and the binding affinities of *Cs*BGL and stevioside complex were also analyzed and visualized with PyMol 1.3.

### Gene Cloning and Heterogeneous Expression

N-terminal signal peptide of the putative β-glucosidase *Cs*BGL was predicted in SignalP-5.0 Server.^6^ The genomic DNA of *C. scophthalmum* 1433 was extracted and used as the template for PCR. The primers were designed based on the gene sequence of *CsBGL* without signal peptides. The sequence of forward and reverse primers were 5′-GGGTCGCGGATCCGAACAGGAAATGGTTACAAAGCC-3′ and 5′-CGAGTGCGGCCGCAAGTTTCGTCCAGTTGATTTT TG-3′; the homologous ends (underlined) were used for the construction of expression plasmid of pET28a-*CsBGL*. The PCR procedures consisted of an initial denaturation step at 94°C for 5 min, 30 replication cycles (94°C for 20 s, 60°C for 15 s, and 68°C for 90 s), and a final extension step at 68°C for 10 min. The PCR products were purified and assembled with the linearized expression plasmid pET28a by T5 Exonuclease, (Xia et al., 2019) and transformed into *E. coli* BL21(DE3). The proper transformants were incubated at 37°C in LB medium with 50 μg/ml kanamycin; when the cell density reached 0.8 at 600 nm, 0.1 mM of isopropyl-β-D-thiogalactopyranoside (IPTG) was added to induce the expression of recombinant *Cs*BGL. After continuous cultivation for 5 h, the cells were harvested and lysed by sonication. The lysate was centrifuged at 15,000 × *g* for 15 min at 4°C and the supernatant was subjected to enzyme purification through nickel affinity chromatography. The purified protein samples were detected by 10% (w/v) of sodium dodecyl sulfate polyacrylamide gel electrophoresis (SDS-PAGE), (Brunelle and Green, 2014) and proteins in the gel were visualized by CBB R-250 staining.

### Enzyme Assays

The β-glucosidase activity was measured by mixing 5 μl enzyme solution with 45 μl 2 mM *p*NP-β-Glc in 50 mM sodium phosphate buffer (pH 7.4) and then incubated at 45°C for 5 min. The reaction was stopped by addition of 100 μl 1 M Na_2_CO_3_ solution. The liberated *p*-nitrophenol was measured at 405 nm by a Synergy LX microplate reader (BioTek, United States) in 96-well microtiter plates. One unit of enzyme activity (U) was defined as the amount of enzyme required to liberate 1 μmol *p*-nitrophenol per minute under the assay condition. Activity assays were also performed for other nitrophenyl glycosides under the same conditions, including *p*NP-β-Glc, *p*NP-β-GlcNAc, *p*NP-β-GlcA, *p*NP-β-Gal, *p*NP-β-Fuc, *p*NP-β-Man, *p*NP-α-Glc, and *p*NP-α-Gal. Protein concentration was determined according to the Bradford assay protocol (Zuo and Lundahl, 2000), with bovine serum albumin (BSA) as standard.

### Biochemical Characterization

The substrate specificity of *Cs*BGL was determined by incubating the enzyme (0.5 U/ml) with 5 mM various substrates under the enzyme assay conditions. The reaction was terminated by boiling for 5 min and the released glucose was determined by Glucose Assay Kit with glucose as a standard. The hydrolysis products from steviol glucosides were determined by high performance liquid chromatography (HPLC).

The optimal pH of *Cs*BGL was determined by measuring the enzyme activity with *p*NP-β-Glc at gradient pH values ranging from 3.5 to 8.0 in 100 mM sodium hydrogen phosphate-citric acid buffer, 8.0–9.0 in 50 mM Tris–HCl buffer, and 9.0–10.5 in 50 mM glycine-sodium hydroxide buffer. The pH stability of *Cs*BGL was determined by incubating the enzyme in the presence of the above various buffers at 4°C for 24 h and measuring the residual enzyme activity. The optimal temperature of *Cs*BGL was determined by measuring the enzyme activity with *p*NP-β-Glc at gradient temperatures from 30 to 50°C (2.5°C intervals) for 5 min. The temperature stability of *Cs*BGL was determined by incubating the enzyme at 40, 42.5, and 45°C for 15 min to 240 min and measuring the residual enzyme activity. Furthermore, the effects of metal ions including AgNO_3_, KCl, LiCl, HgCl_2_, CuSO_4_, CrSO_4_, MnCl_2_, CaCl_2_, NiSO_4_, FeCl_2_, FeCl_3_, CoSO_4_, and ZnSO_4_ were explored through determining the enzyme activity in the presence of 2 mM metal salts. All the experiments were carried out in triplicate.

The initial reaction velocity was measured by incubating *Cs*BGL (0.77 U/ml) in 50 μl 50 mM sodium phosphate buffer (pH 7.4) containing 0, 20, 40, and 80 mM glucose and 0.1–10 mM *p*NP-β-Glc under the enzyme assay conditions and the enzyme activity was examined. *K*_*m*_, *k*_cat_, and *K*_*i*_ were calculated in GraphPad Prism 8 software. All the experiments were carried out in triplicate.

### Production and Isolation of Rubusoside

The conditions to produce rubusoside from stevioside were explored using 600 μg/ml (30.9 U/ml) enzyme and 240 g/L stevioside. The effects of pH values were examined at 45°C for 40 min at various pH values from 5 to 8.0 in 100 mM sodium hydrogen phosphate-citric acid buffer, 8.0–9.0 in 50 mM Tris–HCl buffer, and 9.0–10 in 50 mM glycine-sodium hydroxide buffer. The effects of temperature were examined in 50 mM sodium phosphate buffer (pH 6.5) for 40 min at different temperatures ranging from 35 to 50°C with 2.5°C intervals. The time-course analysis was performed in 1 ml 50 mM sodium phosphate buffer (pH 6.5) at 47.5°C for 5–120 min, and the sample (50 μl) was taken out with 5–10-min intervals. Using the crude steviol glycosides extract as the substrate, we used 1 L reaction mixture containing 500 g crude steviol glycosides extract and 300 μg/ml (15.5 U/ml) *Cs*BGL in 50 mM sodium phosphate buffer (pH 6.5) at 47.5°C for 2 h. The reaction was stopped by boiling for 5 min, and the glucose released was detected using the Glucose Assay Kit, and the hydrolysis products were identified by HPLC analysis.

Rubusoside was isolated by gel filtration chromatography on a Bio-Gel P2 column (3.5 × 120 cm) with distilled water as the eluent. The eluted fractions were collected and monitored by TLC, and the identified fractions were combined and concentrated by evaporation and lyophilized to dry powder.

The conversion of stevioside (molar ratio) was calculated by dividing the concentration of converted stevioside by its initial concentration. The yield of rubusoside (molar ratio) was calculated through dividing the concentration of produced rubusoside by the initial concentration of stevioside.

### High Performance Liquid Chromatography and Thin Layer Chromatography Analysis

HPLC was performed by an Agilent 1260 series coupled with a UV detector (G1314B) using a C18 analysis column (Agilent poroshell 120 EC-C18, 150 × 4.6 mm, 4 μm) at 40°C. Samples were detected by the UV absorbance at 210 nm. The binary mobile phase included solvent A (10 mM KH_2_PO_4_ solution in water, 0.1% TFA) and solvent B (acetonitrile, 0.1% TFA) at a flow rate of 0.4 ml/min for 35 min. The acetonitrile concentrations were changed as follows: 36% (1–7 min), 36–80% (7–12 min), 80% (12–16 min), 80–90% (16–17 min), 90% (17–20 min), 90–36% (20–25 min), after running 10 min (Wang et al., 2021).

TLC was performed by loading samples on silica gel 60 F254 plates (Merck, Germany). The developing solvent was a mixture of chloroform/methanol/water (8:5:1, v/v/v) (Richman et al., 2005). The chromogenic reagent was 0.5% (w/v) 3,5-dihydroxytoluene in 20% (v/v) sulfuric acid. The chromogenic condition was heating at 125°C for 4 min.

### Mass Spectrometry and Nuclear Magnetic Resonance

The MS analysis of steviol glycosides including RD, RA, stevioside, rubusoside, steviolbioside, and steviolmonoside were performed through Shimadzu LCMS-IT-TOF (Kyoto, Japan) equipped with an ESI source in negative ion mode at a resolution of 10,000 full width at half-maximum. The NMR spectra of rubusoside was recorded on Agilent DD2-600 spectrometer at 150 MHz for ^13^C and 600 MHz for ^1^H in the mixture of DMSO-d_6_ (600 μL) and D_2_O (20 μL) at 25°C. Chemical shifts were expressed in parts per million (ppm) downfield from the internal tetramethylsilane of DMSO-d_6_. Homonuclear ^1^H/^1^H correlation experiments (COSY and TOCSY) and heteronuclear ^1^H/^13^C correlation experiments (HSQC and HMBC) were run using the standard pulse sequences.

## Results

### Isolation and Identification of a Stevioside-Hydrolyzing Microorganism

Only strain 1433 was isolated from the soil samples using the solid and liquid selective media with stevioside as the sole carbon source. Its pure culture showed yellow, opaque, round, and convex on LB agar plate (Supplementary Figure 1A). The cells were gram-negative, rod-shaped, non-motile, and non-spore-forming when observed under the microscope (Supplementary Figure 1B). The draft genome of strain 1433 contains 4,324,461 bp with the G + C ratio of 33% (GenBank no. JAFLHF000000000.1). In total, there were 4,043 coding sequences (CDSs), 65 tRNA genes, and 6 rRNA genes in its genome. Specifically, 52 glycoside hydrolases, 67 glycosyltransferases, 8 polysaccharide lyases, and 49 carbohydrate esterases were predicted according to the gene annotation. The whole-genome-based evolutionary tree of multiple species of *Chryseobacterium* showed that the isolated strain 1433 is closest to *C. scophthalmum* DSM16779 and next closest to *C. scophthalmum* VV8 (Supplementary Figure 1C). The 16S rRNA of strain 1433 (GenBank no. MW689256.1) showed a sequence identity of 99.26% with the type strain of *C. scophthalmum* Cl-20 (GenBank no. KC178594.1). All the results suggest that strain 1433 was *C. scophthalmum*.

### Cloning and Heterogeneous Expression of *Cs*BGL

The analysis of the hydrolysis of stevioside showed that the cells and the soluble-lysate could hydrolyze stevioside while the supernate of the culture could not (Supplementary Figure 2), which indicated that the functional enzyme might mainly exist in cells. In the predictive proteome of *C. scophthalmum* 1433, we found four putative β-glucosidases. To identify the enzyme for hydrolysis of stevioside, we employed native acidic PAGE and active staining to determine the β-glucosidase activity of crude enzymes induced by selection medium (Supplementary Figure 3). Subsequently, mass spectrum was used to identify the sequence of β-glucosidases by searching against the predictive proteome of *C. scophthalmum* 1433. The 276 peptides of the brown band in active staining were identified by MS, and 37 unique peptides of the MS results matched the putative β-glucosidase protein (GenBank no. WP_210150222.1, named *Cs*BGL) with 54.45% coverage. The gene encoding *Cs*BGL protein was 2,328 nucleotides consisting of 775 amino acids with a predicted molecular mass of 84.7 kDa and an isoelectric point of 8.2. By searching the sequence of *Cs*BGL in the Conserved Domains database of NCBI, it was inferred that *Cs*BGL belonged to glycoside hydrolase family 3 (GH3). A signal peptide of 18 amino acid residues in the N-terminal was predicted by SignalP-5.0 Server.

**FIGURE 1:**
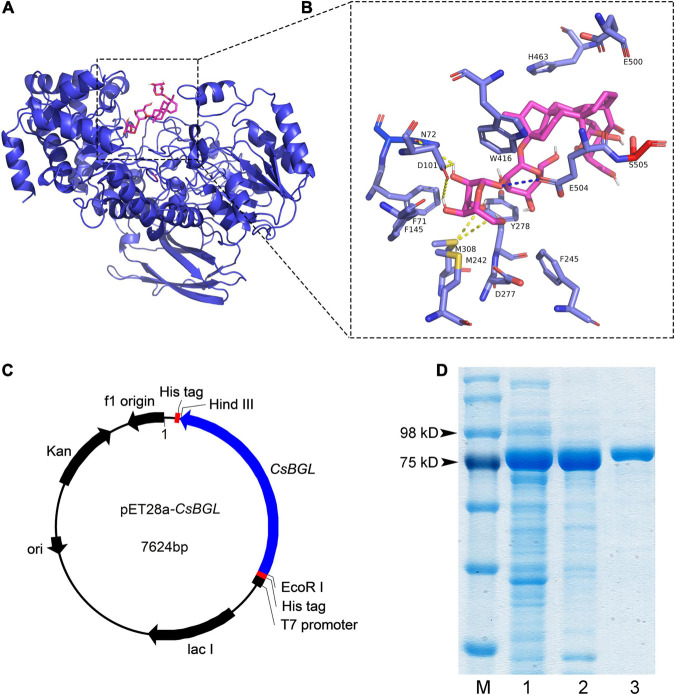
Structure modeling analysis of *Cs*BGL and the heterologous expression of the *Cs*BGL. **(A)** The putative 3D structure of *Cs*BGL (blue) with stevioside (pink). The structure of *Cs*BGL was modeled using *Bt*BGL (PDB entry 5xxn) from *Bacteroides thetaiotaomicron* as the template. **(B)** *Cs*BGL-stevioside interactions. Hydrogen bonds are shown in yellow dotted lines. The unique interaction between E504 (the general acid/base) and glycosidic oxygen is shown in blue dotted line. **(C)** The map of constructed plasmid of *p*ET28a-*Cs*BGL. The cloned gene of *Cs*BGL has 2271 nucleotides without the signal peptide and termination codon. It was inserted between *Hin*dIII and *Eco*RI of *p*ET28a plasmid. **(D)** SDS-PAGE analysis of *Cs*BGL: lane M, protein marker; lane 1, the soluble-lysate after centrifugation; lane 2, the lysate precipitation; lane 3, purified recombinant *Cs*BGL.

Multiple sequence alignment of *Cs*BGL with three other reported β-glucosidases with stevioside hydrolysis activity, including BGL1 from *Streptomyces* sp. *GXT6* CGMCC 7091, SPBGL1 from *S. elodea* ATCC 31461, and EcBgl from *E. casseliflavus* strain EC20, and three well-studied β-glucosidases of GH3, including JMB19063 from a compost metagenome, *Bt*BGL from *B. thetaiotaomicron*, and *Li*BGL from *L. innocua*, was performed. The results (Supplementary Figure 4) showed that *Cs*BGL employed a conserved aspartate residue (D277) as the catalytic nucleophile and a conserved glutamate residue (E504) as the acid/base residue, which was consistent with the general mechanism of GH3 glucosidases. The full-length amino acid sequence of *Cs*BGL only shared 40.10, 41.82, and 8.50% identity with SPBGL1 (GH3), EcBgl (GH3), and BGL1 (GH1), respectively. The 3D structure of *Cs*BGL was modeled using *Bt*BGL (PDB entry 5xxn) from *B. thetaiotaomicron* as the template. The molecular docking of *Cs*BGL with the stevioside as the ligand (Figures 1A,B) showed that N72, D101, and M308 were very important for the binding of stevioside to the enzyme active site. Moreover, E504, the putative catalytic residue as the general acid/base, was precisely oriented to offer the proton for the hydrolysis of the glycosidic bond in the sophorosyl disaccharide of stevioside. The sequence and docking analysis demonstrated that *Cs*BGL was a new β-glucosidase and could specifically hydrolyze stevioside, respectively.

Then, we successfully constructed a plasmid of *p*ET28a-*CsBGL* using the signal-deleted gene of 2,271 nucleotides (Figure 1C) and expressed *Cs*BGL in *E. coli* BL21(DE3). As shown in Figure 1D, in SDS-PAGE, the purified *Cs*BGL migrated as a single protein band with a molecular mass of approximately 86.3 kDa, consistent with the molecular mass prediction of *Cs*BGL without signal peptide (82.8 kDa) which fused with the histidine tag (about 3.5 kDa).

*Cs*BGL showed the highest activity to *p*NP-β-Glc (51.5 U/mg). *Cs*BGL also hydrolyzed *p*NP-β-Gal, *p*NP-α-Glc, and *p*NP-α-Gal with less than 2% relative activity of the *p*NP-β-Glc. No activity was detected for *p*NP-β-GlcNAc, *p*NP-β-GlcA, *p*NP-β-Fuc, and *p*NP-β-Man.

**FIGURE 2:**
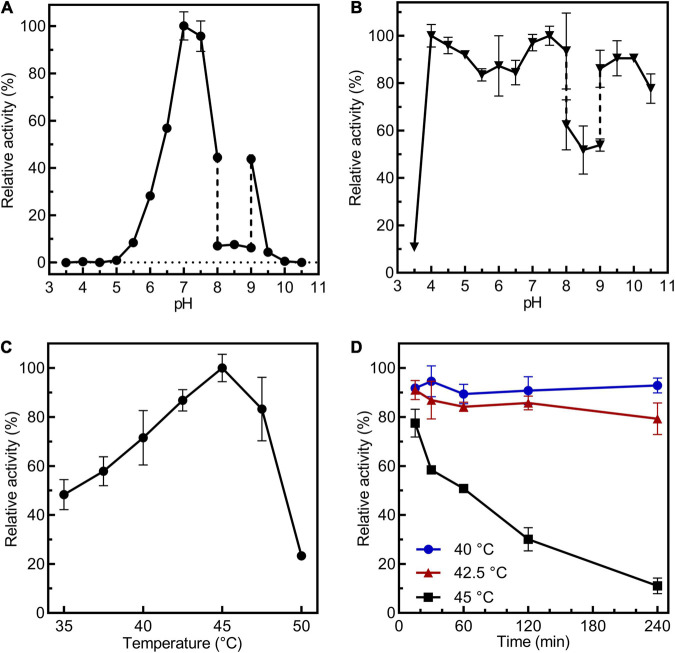
Biochemical characterization of *Cs*BGL. **(A)** Measurements of optimum pH of *Cs*BGL. Reactions were performed at 45°C for 5 min at various pH values from 3.5 to 10.5 using *p*NP-β-Glc as substrate. **(B)** Measurements of pH stability of *Cs*BGL. The *Cs*BGL was incubated in the presence of various pH values from 3.5 to 10.5 at 4°C for 24 h and measuring the residual activity. **(C)** Measurement of optimum temperature of *Cs*BGL. Reactions were performed in 100 mM sodium hydrogen phosphate-citric acid buffer (pH 7.0) with *p*NP-β-Glc at gradient temperatures from 30 to 50°C (2.5°C intervals) for 5 min. **(D)** Measurement of temperature stability of *Cs*BGL. The *Cs*BGL was incubated at 40, 42.5, and 45°C for 15 min to 240 min and measuring its residual activity. All the experiments were carried out in triplicate.

*Cs*BGL retained potent activity at pH 7.0–7.5 and it was stable at pH 4.0–10.0 (Figures 2A,B). *Cs*BGL showed the best activity at 45°C and it showed stability at 40 and 42.5°C, but approximately half of the activity was lost in an hour at 45°C (Figures 2C,D). Ag^+^ drastically inhibited the enzyme activity by 91%. K^+^, Li^+^, Hg^2+^, Cu^2+^, Cr^2+^, Mn^2+^, and Ca^2+^ also impaired the activity by 6–34%. By contrast, Ni^2+^, Fe^2+^, Fe^3+^, Co^2+^, and Zn^2+^ enhanced the activity of *Cs*BGL by 8–28% (Supplementary Figure 5).

Using the *p*NP-β-Glc as substrate, the initial reaction velocity of *Cs*BGL was measured, and the *K*_*m*_, *V*_max_, *k*_cat_, *k*_cat_/*K*_*m*_, and *K*_*i*_ (glucose) values were 1.62 mM, 11.45 μM⋅min^–1^⋅mg^–1^ protein, 16.77 s^–1^, 10.35 s^–1^⋅mM^–1^, and 16.36 mM, respectively (Supplementary Figure 6).

### Determination of the Substrate Specificity of *Cs*BGL for Hydrolysis

**FIGURE 3:**
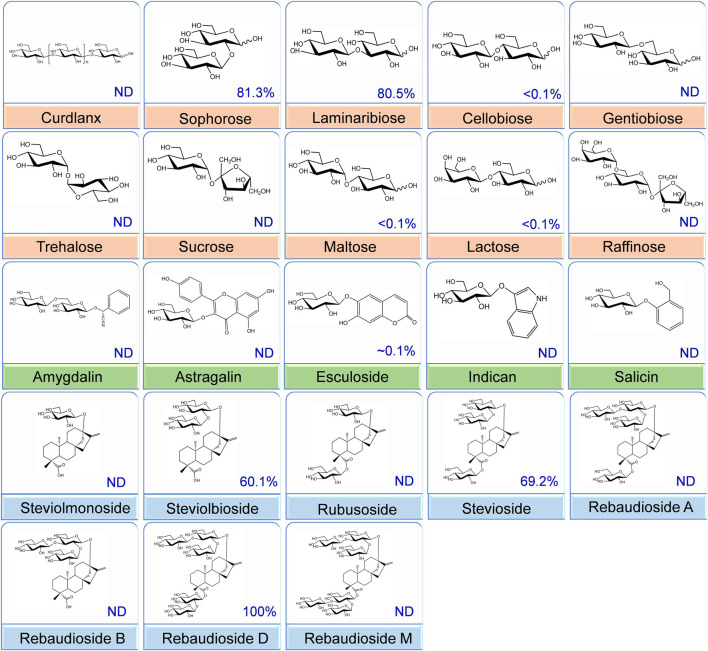
Substrate specificity of the *Cs*BGL. Reactions were performed by incubating the *Cs*BGL (0.5 U/ml) with 5 mM various substrates at 45°C for 5 min. The relative activity (%) with different substrates is given. “ND” indicates that no activity was detected.

To determine the glucosidic linkage specificity of *Cs*BGL, 23 glucosides, including α/β-glucosyl-linkage di-/tri-saccharides, natural products, and some steviol glycosides, were used to measure its catalytic activity (Figure 3). *Cs*BGL showed the highest activity to RD with the C-13-linked Glcβ1-2[Glcβ1-3]Glc trisaccharide and the C-19-linked Glcβ1-2Glc disaccharide (sophorese). Compared with the hydrolysis of RD, *Cs*BGL displayed the relative activities ranging from 60.10 to 81.34% to stevioside (69.2%) with C-13-linked Glcβ1-2Glc disaccharide (sophorese) and the C-19-linked Glcβ1-monosaccharide, steviolbioside (60.1%) with C-13-linked Glcβ1-2Glc disaccharide (sophorese) and the C-19-linked hydroxy group, sophorose (81.3%, Glcβ1-2Glc) and laminaribiose (80.5%, Glcβ1-3Glc). By contrast, the hydrolysis activity of *Cs*BGL to cellobiose (Glcβ1-4Glc), gentiobiose (Glcβ1-6Glc), maltose (Glcα1-4Glc), lactose (Galβ1-4Glc), and esculoside (Glcβ-aglycon) was poor with relative activities of ∼0.1% or lower than 0.1%. Notably, *Cs*BGL could not hydrolyze rubusoside with the C-13 or/and C19-linked Glcβ1-monosaccharides, steviolmonoside with the C-13-linked Glcβ1- monosaccharide, and the C-19-linked hydroxy group, RA with the C-13-linked Glcβ1-2[Glcβ1-3]Glc trisaccharide and the C-19-linked Glcβ1-monosaccharide, RB with the C-13-linked Glcβ1-2[Glcβ1-3]Glc trisaccharide and the C-19-linked hydroxy group, RM with the C-13 and C19-linked Glcβ1-2[Glcβ1-3]Glc trisaccharides, curdlan (Glcβ1-3Glc glucans), sucrose (Glcα1-2βFru), trehalose (Glcα1-1αGlc), raffinose (Galα1-6Glcα1-2βFru), astragalin (Glcβ-aglycon), amygdalin (Glcβ1-6Glcβ-aglycon), indican (Glcβ-aglycon), and salicin (Glcβ-aglycon).

**FIGURE 4:**
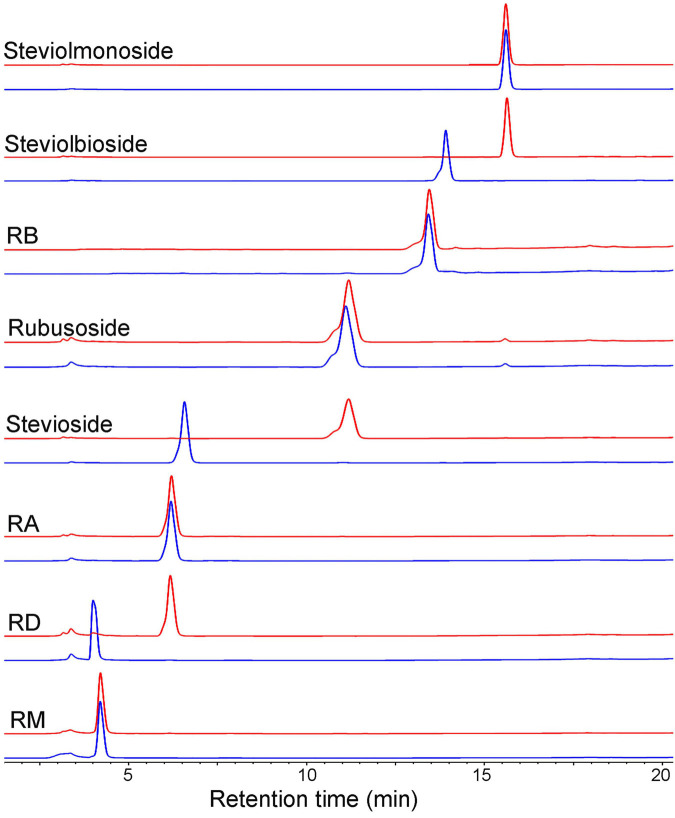
HPLC analysis of the hydrolysis products of steviol glucosides by *Cs*BGL. Reactions were performed by incubating *Cs*BGL (0.5 U/ml) with 5 mM RM, RD, RA, stevioside, rubusoside, RB, steviolbioside, and steviolmonoside in the 50 mM sodium phosphate buffer (pH 7.4) at 45°C for 5 min. The red lines represent the hydrolysis product of steviol glycosides by *Cs*BGL and the blue lines represent the control of steviol glycosides.

Next, we employed HPLC (Figure 4) and ESI-MS (Supplementary Figure 7) to determine the hydrolysis products of different steviol glucosides by *Cs*BGL. RD was hydrolyzed to RA with the retention time at 6.2 min in HPLC and the peak of [M-H]^–^ ion at *m/z* 965.41, consistent with the molecular mass of RA (966.43). Stevioside was hydrolyzed to rubusoside with the retention time at 11.2 min in HPLC and the peak of [M-H]^–^ ion at *m/z* 641.32, consistent with the molecular mass of rubusoside (642.32). Steviolbioside was hydrolyzed to steviolmonoside with the retention time at 15.6 min in HPLC and the peak of [M-H]^–^ ion at *m/z* 479.26, consistent with the molecular mass of steviolmonoside (480.27). Notably, the results of HPLC also showed that RM, RA, RB, rubusoside, and steviolmonoside were not hydrolyzed, which agreed with the biochemical measurements. Collectively, these results suggested that when steviol glucosides were used as substrates, *Cs*BGL specifically cleaved the C-13 or C-19-linked β-1,2-glucosidic linkage in sophorosyl disaccharide, whereas it could not hydrolyze a single β-D-glucopyranosyl unit and the Glcβ1-2[Glcβ1-3]Glc trisaccharide moiety.

### The Mass Production of Rubusoside From Stevioside

**FIGURE 5:**
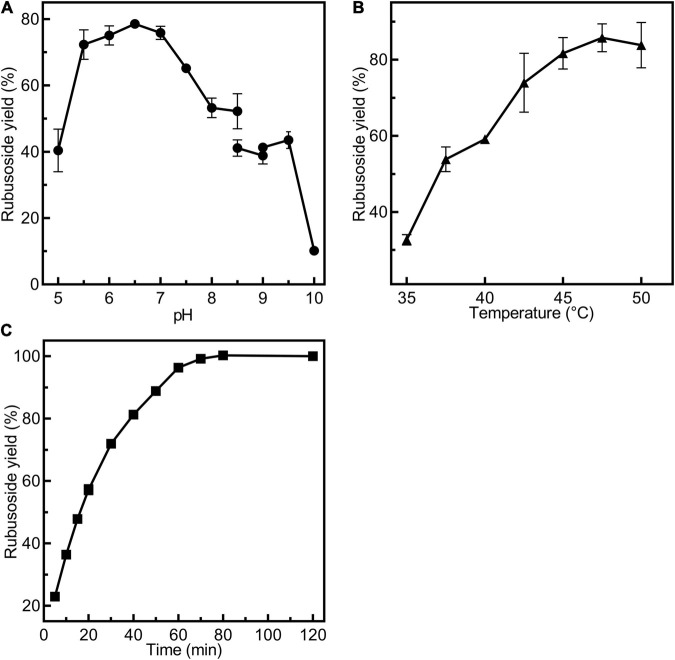
Condition exploration for production of rubusoside using 30.9 U/ml *Cs*BGL and 240 g/L stevioside. **(A)** Effects of pH on the rubusoside yield. Reactions were performed at 45°C for 40 min at various pH values from 5 to 10. **(B)** Effects of temperature on the rubusoside yield. Reactions were performed in 50 mM sodium phosphate buffer (pH 6.5) for 40 min at several different temperatures ranging from 35 to 50°C with 2.5°C intervals. **(C)** The time course of rubusoside yield from 5 to 120 min. Reactions were performed in 1 ml 50 mM sodium phosphate buffer (pH 6.5) at 47.5°C for 5 to 120 min. A 50 μl sample was taken out with 5–10-min intervals. All the experiments were carried out in triplicate.

The reactions for production of rubusoside were performed by incubating *Cs*BGL (600 μg/ml, 30.9 U/ml) with stevioside (240 g/L). The pH value strongly affected the rubusoside yield at 45°C for 40 min. As showed in Figure 5A, the rubusoside yield increased from 40.4% at pH 5.0 to 78.5% at pH 6.5 and then decreased to 10.1% at pH 10.0. The reaction temperature also markedly affected the hydrolysis of stevioside at pH 6.5 for 40 min. When the temperature increased from 35 to 47.5°C, the rubusoside yield increased from 32.6% at 35°C to the maximum yield of 85.7% at 47.5°C, then the yield slightly decreased to 83.8% at 50°C (Figure 5B). The time course of the reaction at 47.5°C and pH 6.5 showed that the highest stevioside conversion of 99% and the rubusoside yield of 99% were achieved at 70 min (Figure 5C).

The initial reaction velocity of *Cs*BGL for the hydrolysis of stevioside was also determined in 50 mM sodium phosphate buffer (pH 6.5) at 47.5°C. The *K*_*m*_, *V*_max_, *k*_cat_, and *k*_cat_/*K*_*m*_ values were 1.54 mM, 169.70 μM⋅min^–1^⋅mg^–1^ protein, 248.59 s^–1^, and 160.49 s^–1^⋅mM^–1^, respectively (Supplementary Figure 8).

**FIGURE 6:**
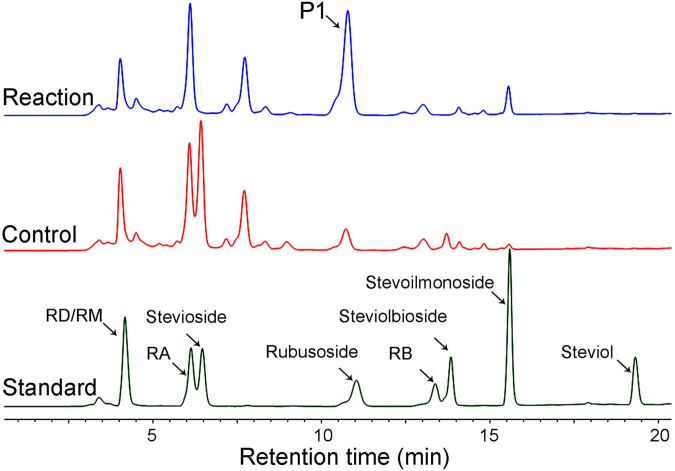
HPLC analysis of the hydrolysis products of crude steviol glycosides extract (500 g/L) by *Cs*BGL. Reactions (blue line) were performed by incubating *Cs*BGL (15.5 U/ml) with crude steviol glycosides extract in 50 mM sodium phosphate buffer (pH 6.5) at 47.5°C for 2 h. P1 indicates rubusoside. The control (red line) was performed by inactive enzyme under the same condition, with the same reactions. The standard substrates are shown in dark green line.

Moreover, using the crude steviol glycosides extract as the substrate, we carried out the rubusoside production by *Cs*BGL; 500 g/L crude steviol glycosides extract containing ∼226 g/L of stevioside (calculated by the peak area of HPLC) and 300 μg/ml (15.5 U/ml) *Cs*BGL was used in the reaction. The HPLC results revealed that almost all stevioside in the crude steviol glycosides extract was hydrolyzed and converted to rubusoside (P1, Figure 6) at 47.5°C for 2 h. The rubusoside concentration was increased from the initial 42 g/L in the crude steviol glycosides extract to 222 g/L in the final reaction solution. At the same time, it was found that RD with the Glcβ1-2Glc sophorese at the C-19 site and steviolbioside with the Glcβ1-2Glc sophorese at the C-13 site were hydrolyzed to RA and steviomonoside, respectively, and another major steviol glycoside RA with a single β-D-glucopyranosyl unit at the C-19 site and the Glcβ1-2[Glcβ1-3]Glc trisaccharide moiety at the C-13-site were not hydrolyzed in the reaction.

### Isolation and Identification of Rubusoside

**FIGURE 7:**
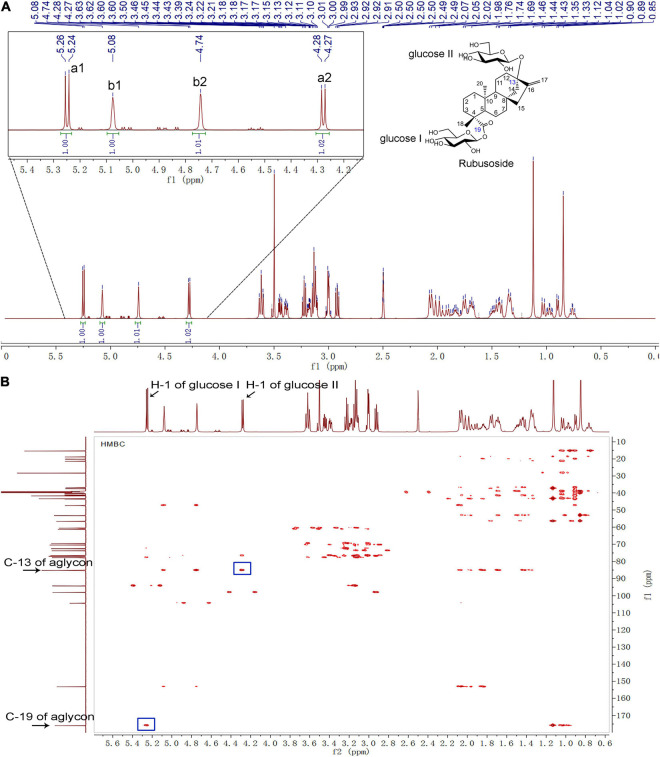
NMR analysis of the hydrolysis production from stevioside by *Cs*BGL. (δ: ppm) **(A)** ^1^H-NMR 600 MHz spectrum. The four peaks in the anomeric region (4.2–5.3 ppm) were labeled as a1, b1, b2, and a2, from low to high field. **(B)** HMBC NMR spectrum. The H-1 of glucose I/II, C-13/19 of aglycon, and their cross peaks were labeled.

**FIGURE 8:**
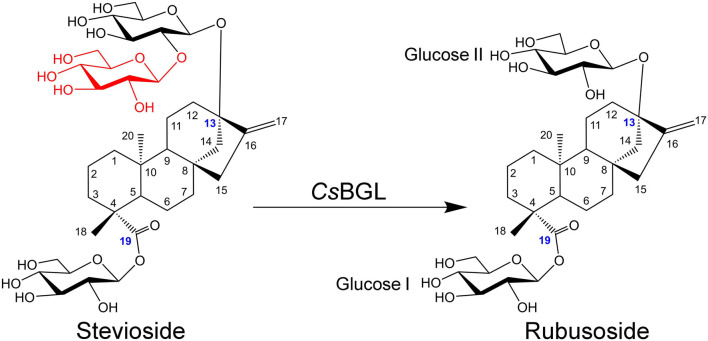
Schematic diagram of production of rubusoside from the hydrolysis of stevioside catalyzed by *Cs*BGL. The blue numbers label the carbon atoms that link with the sugar groups, the different structural moiety between stevioside and rubusoside is displayed in red.

The hydrolysis product from stevioside by *Cs*BGL was separated by Bio-Gel P2 and its chemical structure was identified. In the HPLC analysis, the retention time (11.2 min) of the hydrolysis product was consistent with rubusoside control (Figure 4). The negative-ion ESI-MS analysis of the hydrolysis product showed peaks of [M-H]^–^ ion at *m/z* 641.32, [M + HCOO]^–^ ion at *m/z* 687.32 and [M + CF_3_COO]^–^ ion at *m/z* 755.31; consistent with the molecular mass of rubusoside (642.33), the peak of [M-Glc-H]^–^ ion at *m/z* 479.26 was consistent with a fragment (480.27) of rubusoside, due to the breakdown at the glucose ester bond at the C-19 site (Supplementary Figure 7B). The alkaline hydrolysis analysis showed that the hydrolysis product contained a glucopyranosyl unit at the C-19 site, same as the rubusoside (Supplementary Figure 9). Furthermore, NMR spectrum further confirmed that the hydrolysis product from stevioside by *Cs*BGL was rubusoside. In the full ^1^H NMR spectrum (Figure 7A), four peaks, including δ 5.25 (d, *J* = 8.1 Hz, ^1^H), δ 5.08 (s, ^1^H), δ 4.74 (s, ^1^H), and 4.28 (d, *J* = 7.8 Hz, ^1^H) were detected in the anomeric region (4.2–5.3 ppm) which were labeled as a1, b1, b2, and a2 from low to high field, respectively. The high *J* coupling distances for peaks a1 (8.1 Hz) and a2 (7.8 Hz) suggested that glucose residues of a1 and a2 adopted a β-configuration, while peaks b1 and b2 were single peaks and showed that they were not anomeric proton signals and resolved as the proton signals linked to olefin (C-17) of aglycon. In the ^13^C NMR spectrum, because of the carboxyl group (C-19) and C = C double bonds (C-16 and C-17), three peaks were observed at downfield (C-19, 175.92 ppm; C-16, 153.23 ppm; and C-17, 104.39 ppm) (Supplementary Figure 10). The anomeric proton and carbon of the sugar units were assigned by the ^1^H-^1^H COSY, HSQC, HMBC, and TOCSY (Supplementary Figures 11–13 and Figure 7B). From the HMBC, a cross peak between H-1 of glucose I (δ 5.25 ppm) and C-19 of aglycon (δ 175.92 ppm) was clearly found (Figure 7B), indicating that the glucose I was linked on the C-19 of aglycon. A cross peak between H-1 of glucose II (δ 4.28 ppm) and C-13 of aglycon (δ 85.19 ppm) was clearly found (Figure 7B), indicating that the glucose II was linked to the C-13 of aglycon. Moreover, the complete NMR data are characterized in the Supplementary Material and the chemical shifts of proton and carbon signals of rubusoside are listed in Supplementary Table 1. Accordingly, the chemical structure of the hydrolysis product from stevioside by *Cs*BGL was rubusoside. The NMR spectrum of rubusoside was consisted in the works reported by Guo et al. (2019) and Wang et al. (2021). The process of *Cs*BGL conversion of stevioside into rubusoside is shown in Figure 8.

## Discussion

As an environment-friendly strategy to produce bioactive compounds, glycosidases have been widely used to convert steviol glycosides to produce steviol, steviolmonoside, steviolbioside, rubusoside, and other minor components of steviol glycosides (Nakano et al., 1998; Ko et al., 2013; Chen et al., 2014, 2016; Udompaisarn et al., 2017; Lan et al., 2019). In the present study, we identified a novel β-glucosidase, *Cs*BGL, which showed excellent efficient and specific hydrolysis activity to produce rubusoside from stevioside.

*Cs*BGL showed strict hydrolytic specificity to the β-1, 2-glucosidic bond in the sophorosyl disaccharide without β-1,3-glucosidic bond branch and did not hydrolyze the single β-D-glucopyranosyl unit in steviol glycosides. This strict substrate specificity of *Cs*BGL was speculated to be related to the characteristic 3D structures modeling of the enzyme. The docking model of *Cs*BGL with the stevioside as the ligand showed that the sophorosyl disaccharide of stevioside was nestled in the catalytic cavity of *Cs*BGL formed by four aromatic residues (F71, Y278, F245, and W416). The Glcβ1-2[Glcβ1-3]Glc trisaccharide moiety could not get in this narrow catalytic cavity. For the single β-D-glucopyranosyl unit in steviol glycosides, the W416 and H463 blocked the aglycon of steviol glycosides, probably resulting that the single β-D-glucopyranosyl unit could not get to the appropriate site of catalytic cavity. These results could account for the strict substrate specificity of *Cs*BGL that strongly hydrolyzed stevioside but not rubusoside.

In 2012, it was reported that SSGase (a Sigma-Aldrich’s commercial enzyme) could hydrolyze both stevioside and rubusoside, but its hydrolysis activity to rubusoside was low, only about 29.0% of that to stevioside. When the enzyme activity of SSGase was measured with stevioside and rubusoside as substrates, the enzyme activity for stevioside was 14.5 U, while that for rubusoside was 4.2 U. For the rubusoside production from the hydrolysis of stevioside, SSGase could hydrolyze stevioside (280 mM) to produce rubusoside (193 mM) at 63°C for 7 days, which indicated the rubusoside produced in the reaction solution could be accumulated to a certain extent, but the slow hydrolysis of rubusoside by SSGase could cause its concentration to be lower than that of hydrolyzed stevioside (Ko et al., 2012). In 2015, it was reported that the BGL1 from *Streptomyces* sp. GXT6 CGMCC 7091 hydrolyzed stevioside (10 g/L) to produce rubusoside at 50°C for 6 h with the stevioside conversion rate of 98.2% and the rubusoside yield of 78.8% (w/w) (Wang et al., 2015). As reported in 2019, the SPBGL1 from *S. elodea* ATCC 31461 selectively hydrolyzed purified stevioside (240 g/L) or crude steviol glycosides extract (500 g/L) to produce rubusoside at 45°C for 6 h with the stevioside conversion rate of 98.6% and rubusoside yield of 99% using 600 μg/ml of enzyme (Lan et al., 2019). In the same year, the EcBgl from *E. casseliflavus* strain EC20 was also reported to convert stevioside to rubusoside but the stevioside conversion rate and rubusoside yield were not shown (Boonkaew et al., 2019). It is obvious from the above description that the reactions catalyzed by SSGase, BGL1, and SPBGL1 all took a long time (≥6 h). In the present study, a novel β-glucosidase (*Cs*BGL) was found to have much better catalytic properties. When purified stevioside (240 g/L) was used as the substrate in the reaction, *Cs*BGL selectively hydrolyzed stevioside to produce rubusoside at 47.5°C for 70 min with stevioside conversion rate of 99% and rubusoside yield of 99% using 600 μg/ml (30.9 U/ml) of enzyme. When crude steviol glycosides extract (500 g/L containing stevioside ∼226 g/L) were used as the substrate in the reaction, *Cs*BGL selectively hydrolyzed stevioside, RD, and steviolbioside to produce rubusoside, RA, and steviolmonoside, respectively, at 47.5°C for 2 h with rubusoside yield of ∼99%, using 300 μg/ml (15.5 U/ml) of enzyme. To the best of our knowledge, this is the most efficient enzyme for producing the rubusoside from stevioside. Compared with SPBGL1 from *S. elodea* ATCC 31461 that needs 6 h to catalyze the hydrolysis of purified stevioside (240 g/L) or crude steviol glycosides extract (500 g/L), the reaction time of *Cs*BGL was shortened by about 4.8–4 h with almost the same stevioside conversion and rubusoside yield. The *K*_*m*_, *k*_cat_, and *k*_cat_/*K*_*m*_ values toward stevioside of *Cs*BGL were compared with four other reported β-glucosidases, in particular (Table 1). *Cs*BGL displayed the lowest *K*_*m*_ value of 1.5 mM among the enzymes, which means it had the strongest substrate-binding affinity. Furthermore, the *k*_cat_/*K*_*m*_ value of *Cs*BGL (160.5 s^–1^⋅mM^–1^) was approximately 1.7 and 4.1 times higher than that of the EcBgl (95.38 s^–1^⋅mM^–1^) and SPBGL1 (39.1 s^–1^⋅mM^–1^), respectively, which highlighted its better catalytic performance.

**TABLE 1:** Kinetic parameters toward stevioside of reported β-glucosidase.

**β-Glucosidase**	***K*_*m*_ (mM)**	***k*_cat_ (s^–1^)**	***k*_cat_/*K*_*m*_ (s^–1^mM^–1^)**	**References**
*Cs*BGL	1.5	248.6	160.5	This study
BGL1	1.5	13.2	9.0	Wang et al., 2015
SPBGL1	3.1	121.3	39.1	Lan et al., 2019
SSGase	3.6	30.4	8.5	Ko et al., 2012
EcBgl	1.820	173.60	95.38	Boonkaew et al., 2019

A crude hesperidinase from *Aspergillus niger* (Mizukami et al., 1981) and a β-glucosidase BT_3567 from *B. thetaiotaomicron* HB-13 (Udompaisarn et al., 2017) were used to determine stevioside in *Stevia* samples based on the hydrolysis reaction to release glucose. However, both enzymes could non-specifically hydrolyze RA, which is usually another major component of steviol glycosides, and thereby, the glucose produced by the hydrolysis of RA would affect the accuracy of the determination. Interestingly, *Cs*BGL could specifically cleave the β-1,2-glucosidic linkage in sophorosyl disaccharide of stevioside to produce equimolar glucose without hydrolyzing RA in the crude steviol glycosides extract, which might be developed as a high-throughput method to precisely quantify the stevioside content in *Stevia* samples.

## Conclusion

A novel β-glucosidase *Cs*BGL obtained here could markedly hydrolyze sophorese (Glcβ1-2Glc), laminaribiose (Glcβ1-3Glc), and the C-13- or/and C-19-linked sophorese of steviol glycosides, but not the C-13 or/and C-19-linked Glcβ1-2[Glcβ1-3]Glc trisaccharide and Glcβ1-monosaccharide of steviol glycosides. The enzyme could efficiently convert the high concentration stevioside (240 g/L) at 47.5°C for 70 min and crude steviol glycosides extract (500 g/L) containing ∼226 g/L stevioside at 47.5°C for 2 h to produce rubusoside with the stevioside conversion of 99% and rubusoside yield of 99%. To the best of our knowledge, *Cs*BGL is the most efficient enzyme for the biological production of rubusoside from stevioside and has a potential in industrial applications.

## Data Availability Statement

The datasets presented in this study can be found in online repositories. The names of the repository/repositories and accession number(s) can be found below: https://www.ncbi.nlm.nih.gov/genbank/, https://www.ncbi.nlm.nih.gov/genbank/, https://www.ncbi.nlm.nih.gov/genbank/.

## Author Contributions

ZY designed the research, collected the samples, performed the experiments, analyzed the data, and wrote the manuscript. XC, XY, and SY performed part of the experiments. LX, XJ, and MX supervised the project, analyzed the data, and revised the manuscript. All authors contributed to the article and approved the submitted version.

## Conflict of Interest

The authors declare that the research was conducted in the absence of any commercial or financial relationships that could be construed as a potential conflict of interest.

## Publisher’s Note

All claims expressed in this article are solely those of the authors and do not necessarily represent those of their affiliated organizations, or those of the publisher, the editors and the reviewers. Any product that may be evaluated in this article, or claim that may be made by its manufacturer, is not guaranteed or endorsed by the publisher.

## Footnotes

1
https://github.com/BGI-flexlab/SOAPnuke

2
http://www.ncbi.nlm.nih.gov/BLAST/

3
http://www.clustal.org/

4
https://swissmodel.expasy.org/interactive

5
http://autodock.scripps.edu/

6
http://www.cbs.dtu.dk/services/SignalP/index.php
