# Supplementary material for: A Novel β-Glucosidase From Chryseobacterium scophthalmum 1433 for Efficient Rubusoside Production From Stevioside
Source: Front Microbiol. 2021 Oct 12;12:744914. doi: 10.3389/fmicb.2021.744914 (PMC8546341; doi:10.3389/fmicb.2021.744914)
Supplement: Supplementary file 1 [file Data_Sheet_1.pdf]

Supplementary Material

Supplementary Figures

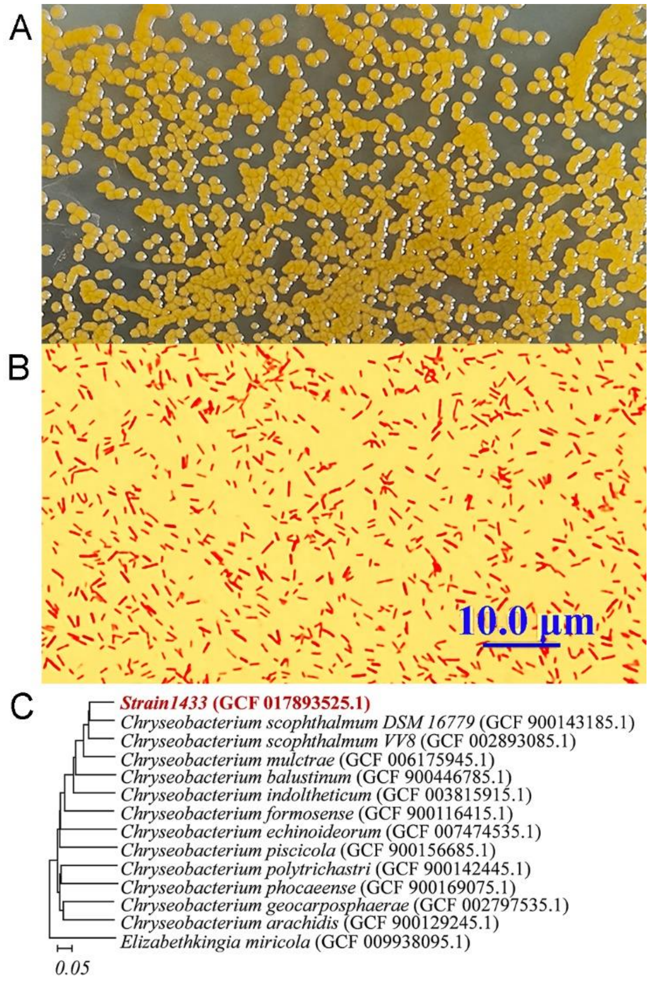

Figure S1. Identification of stevioside-hydrolyzing microorganism. (A) Colony morphology and (B) Gram staining of *C. scophthalmum* 1433. (C) Phylogenetic tree of *Chryseobacterium* bacteria generated by CVTree 3.0 and based on the genomic sequences. The *Elizabethkingia miricola* was used as an outgroup in the phylogenetic trees. The new strain was highlighted in red.

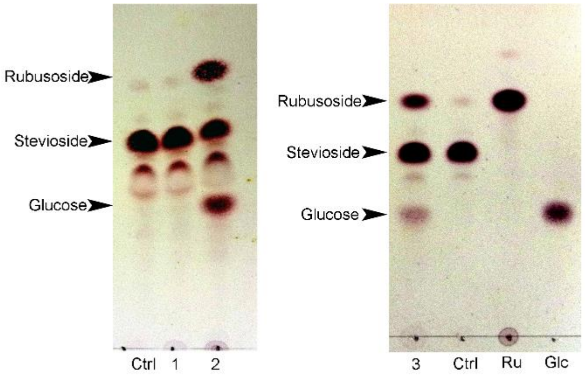

Figure S2. TLC analysis of reaction products with stevioside as the substrate. Line Ctrl, control reaction (inactivated enzymes were used); Line 1, reaction with the culture solution; Line 2, reaction with the cells; Line 3, reaction with the soluble-lysate; Line Ru, standard rubusoside; Line Glc, standard glucose.

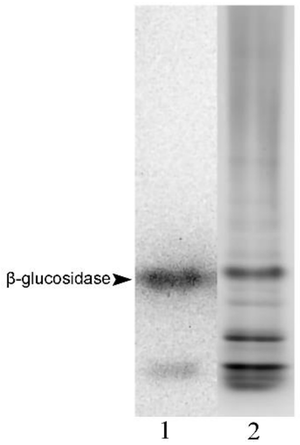

Figure S3. Identification of the novel β-glucosidases. Native acidic PAGE: lane 1, activity-staining. The gel was strained by esculoside (0.5%, w/v) and ferric ammonium citrate (0.05%, w/v) at 40 °C for 15 min; lane 2, CBB-R250 staining.

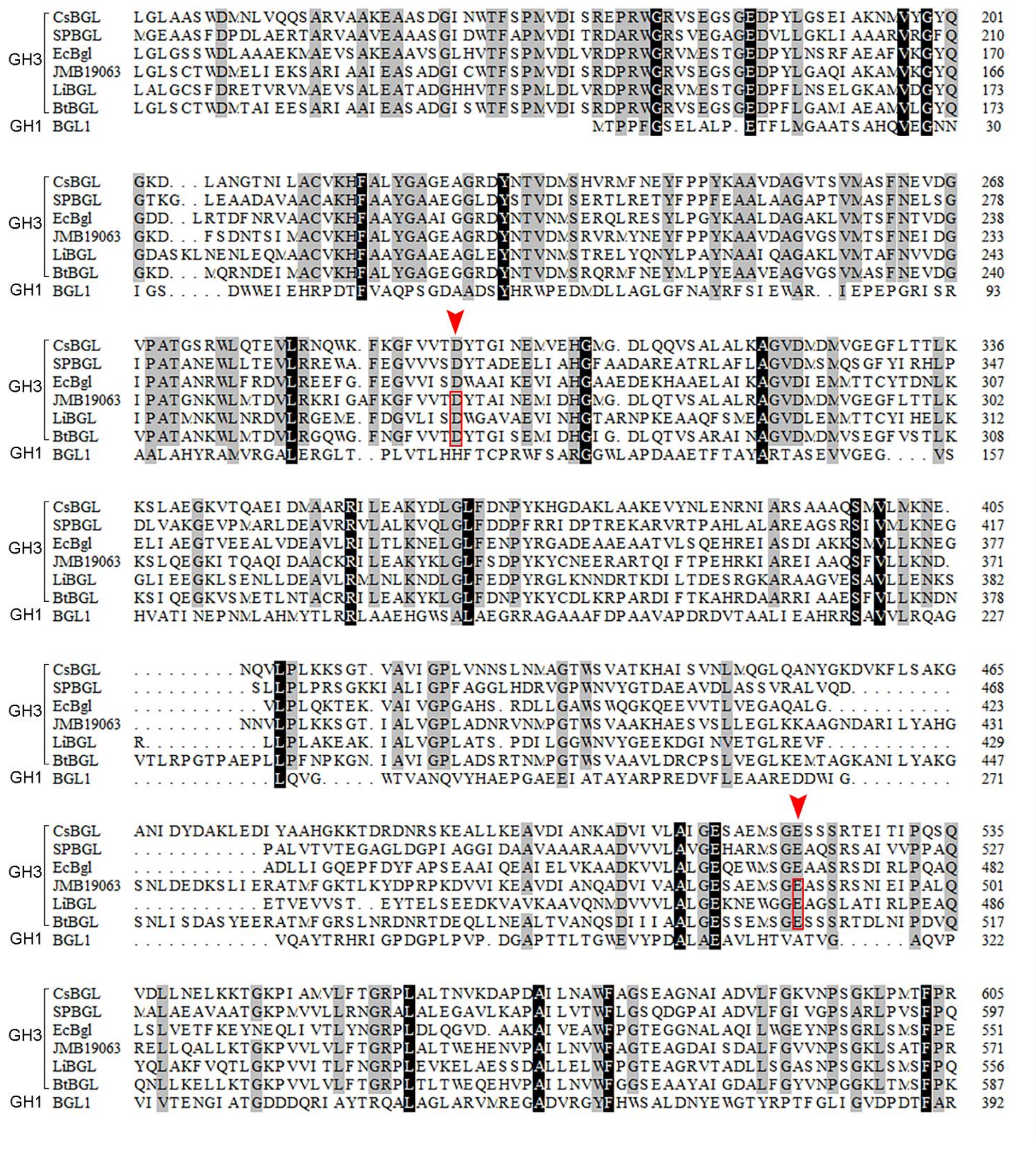

Figure S4. Multiple sequence alignment of *Cs*BGL with three others reported β-glucosidases (SPBGL, EcBGL and BGL1) with stevioside hydrolysis activity and three well studied GH3 β-glucosidases (*Li*BGL, *Bt*BGL and JMB19063). Identical and similar sequence regions are shaded in black and gray, respectively. The conserved nucleophile and acid/base residues are labeled using red arrows, in particular, the catalytic residues that have been experimentally confirmed are marked with red boxes.

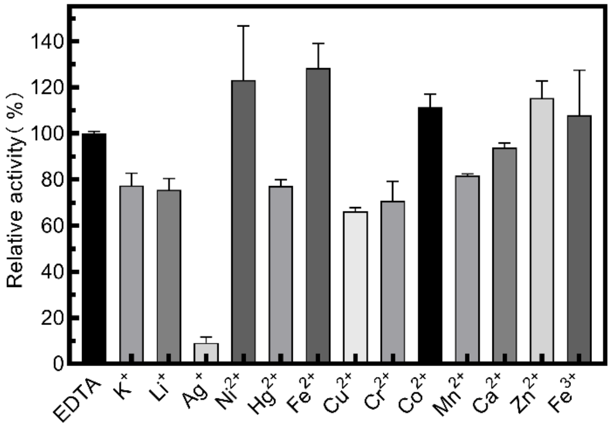

Figure S5. The effects of metal ions on the hydrolysis activity of *Cs*BGL. The reactions were performed in 100 mM sodium hydrogen phosphate-citric acid buffer (pH 7.0) with *p*NP-β-Glc at 45 °C for 5 min in the presence of 2 mM various metal salts. All the experiments were carried out in triplicate.

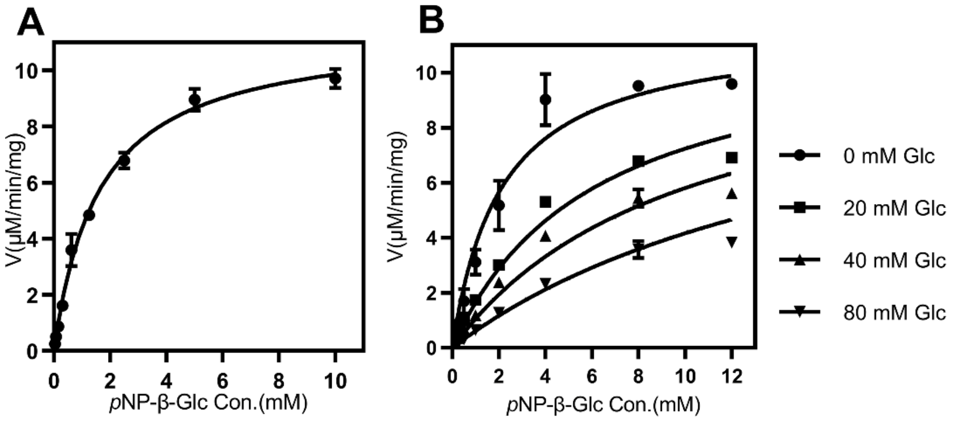

Figure S6. Enzyme kinetic characteristics of *Cs*BGL with *p*NP-β-Glc as substrate. (A) V_{max} and K_m were calculated by Graphpad Prism 8.0.1 using Michaelis-Menten. (B) K_i was calculated by Graphpad Prism 8.0.1 using Competitive inhibition equation (Y = Vmax × X / (Km + X × (1 + X / Ki))). All the experiments were carried out in triplicate.

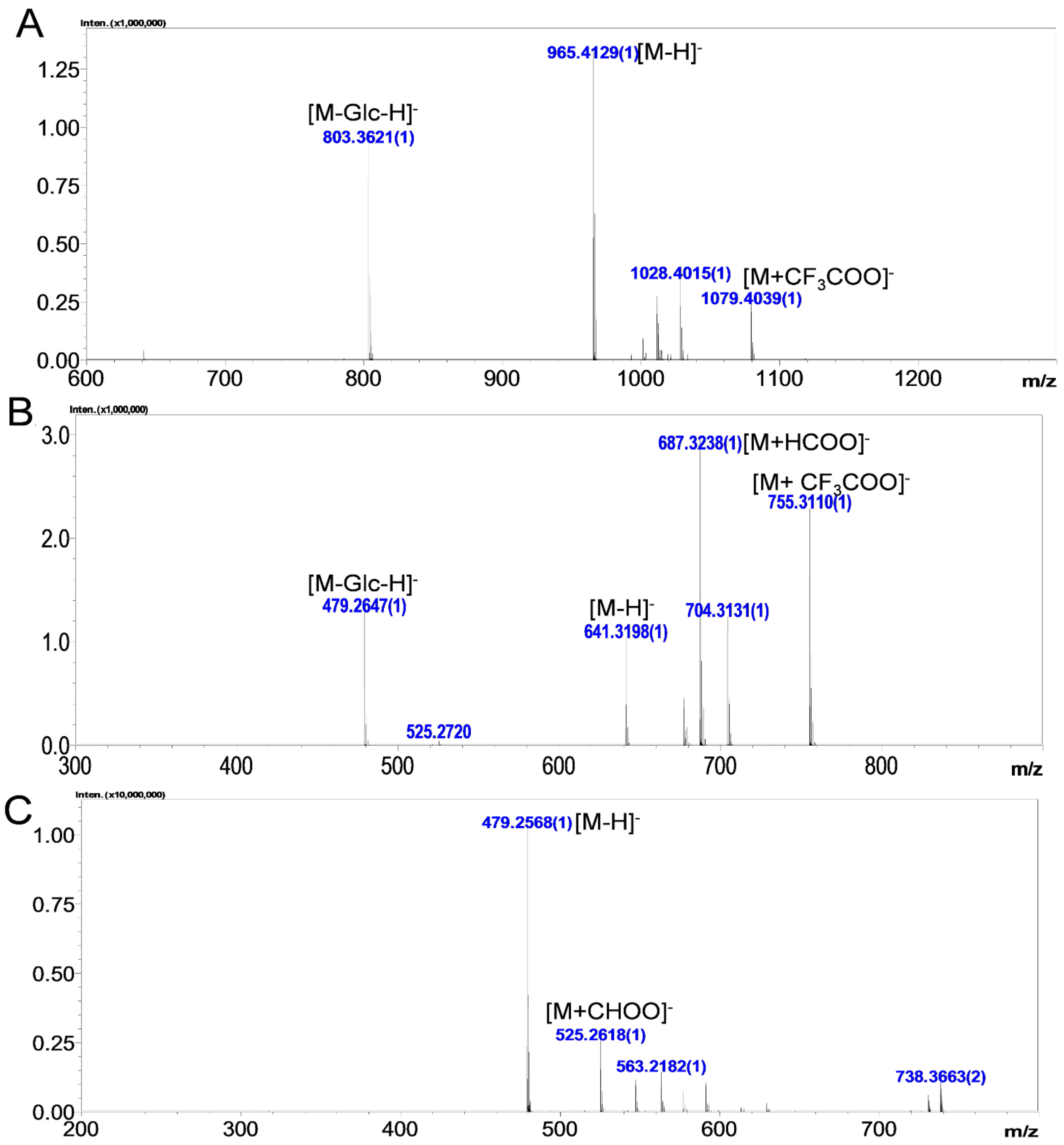

Figure S7. ESI-MS analysis of the hydrolysis reaction of RD, stevioside and steviolbioside catalyzed by the *Cs*BGL. **(A)** MS spectrum of RD reaction; **(B)** MS spectrum of stevioside reaction; **(C)** MS spectrum of steviolbioside reaction.

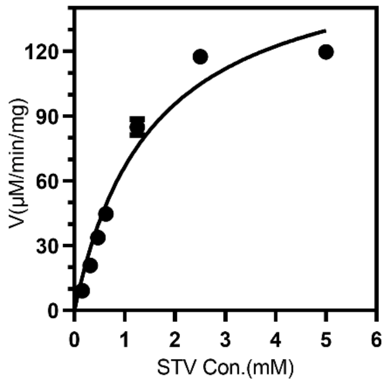

Figure S8. Enzyme kinetic characteristics of *Cs*BGL with stevioside as substrate. V_{max} and K_m were calculated by Graphpad Prism 8.0.1 using Michaelis-Menten. All the experiments were carried out in triplicate.

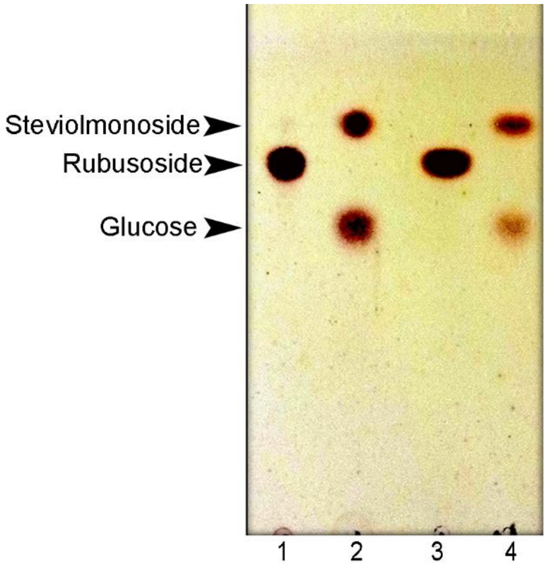

Figure S9. Alkaline hydrolysis analysis of the stevioside hydrolysis product by *Cs*BGL. lane 1, standard rubusoside, lane 2, the alkaline hydrolysis products of the standard rubusoside, lane 3, purified stevioside hydrolysis product, lane 4, the alkaline hydrolysis products of purified stevioside hydrolysis product.

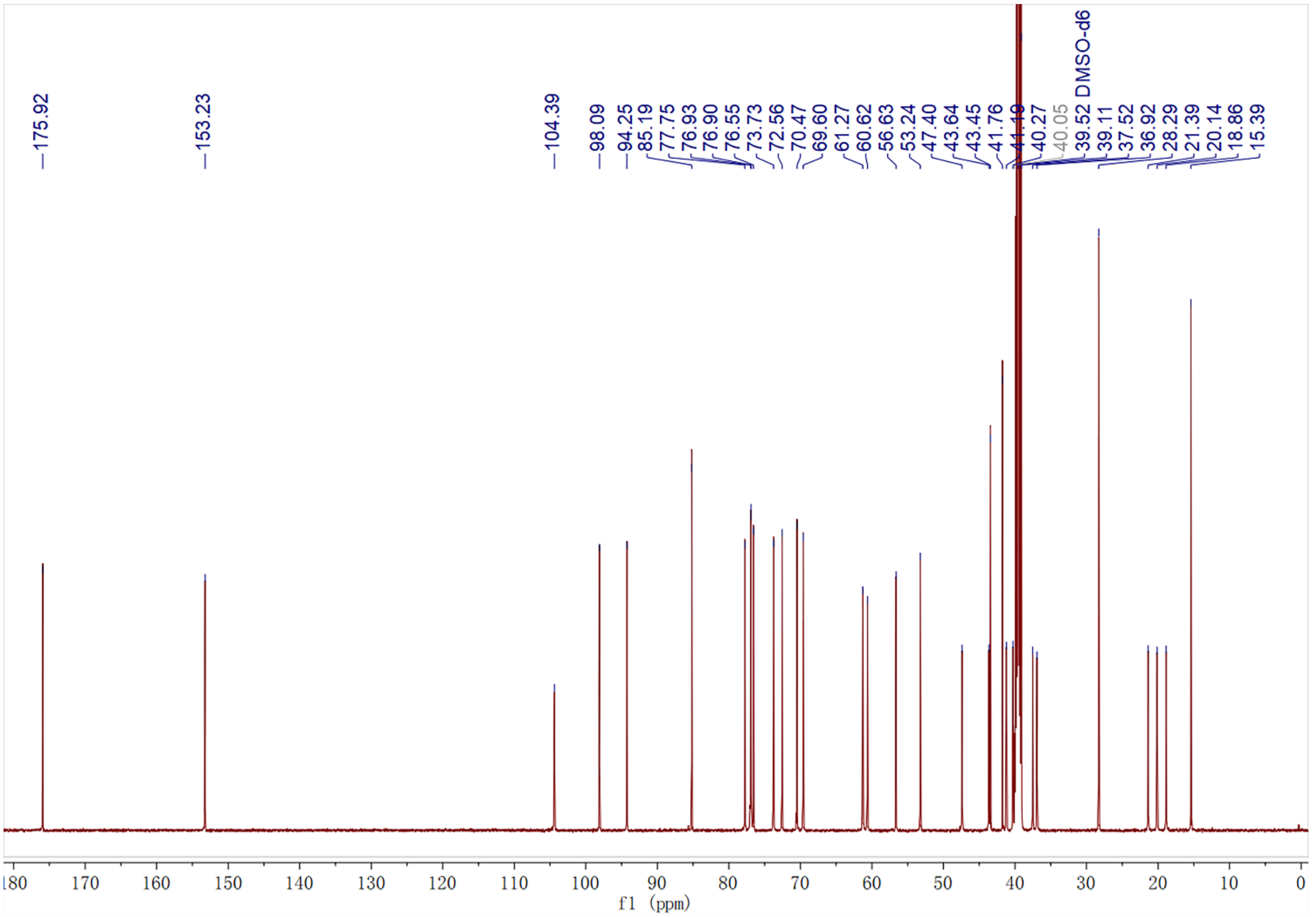

Figure S10. ^{13}C-NMR 150 MHz spectrum of the hydrolysis production from stevioside by *Cs*BGL. (δ: ppm)

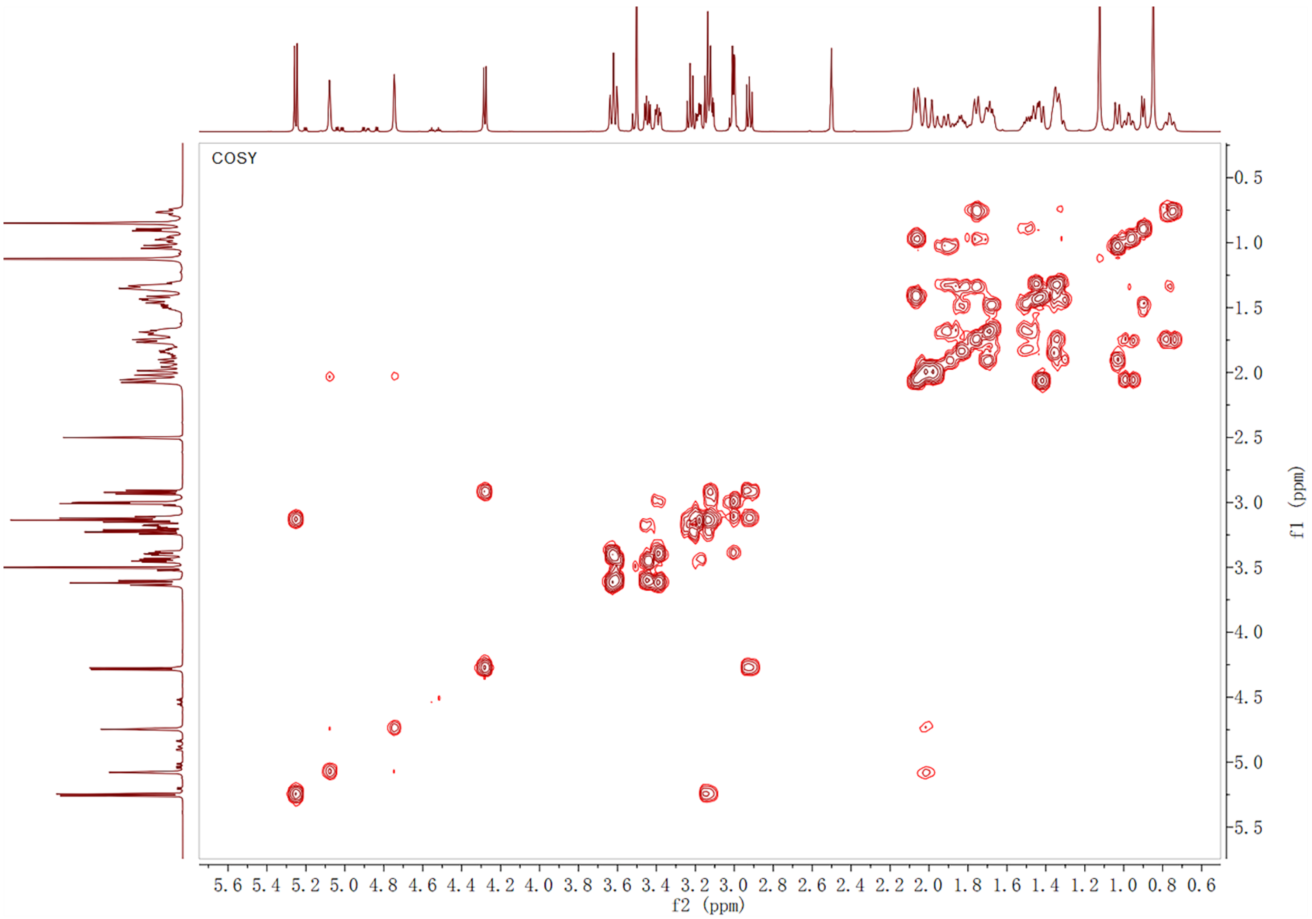

Figure S11. COSY of the hydrolysis production from stevioside by *Cs*BGL. (δ: ppm)

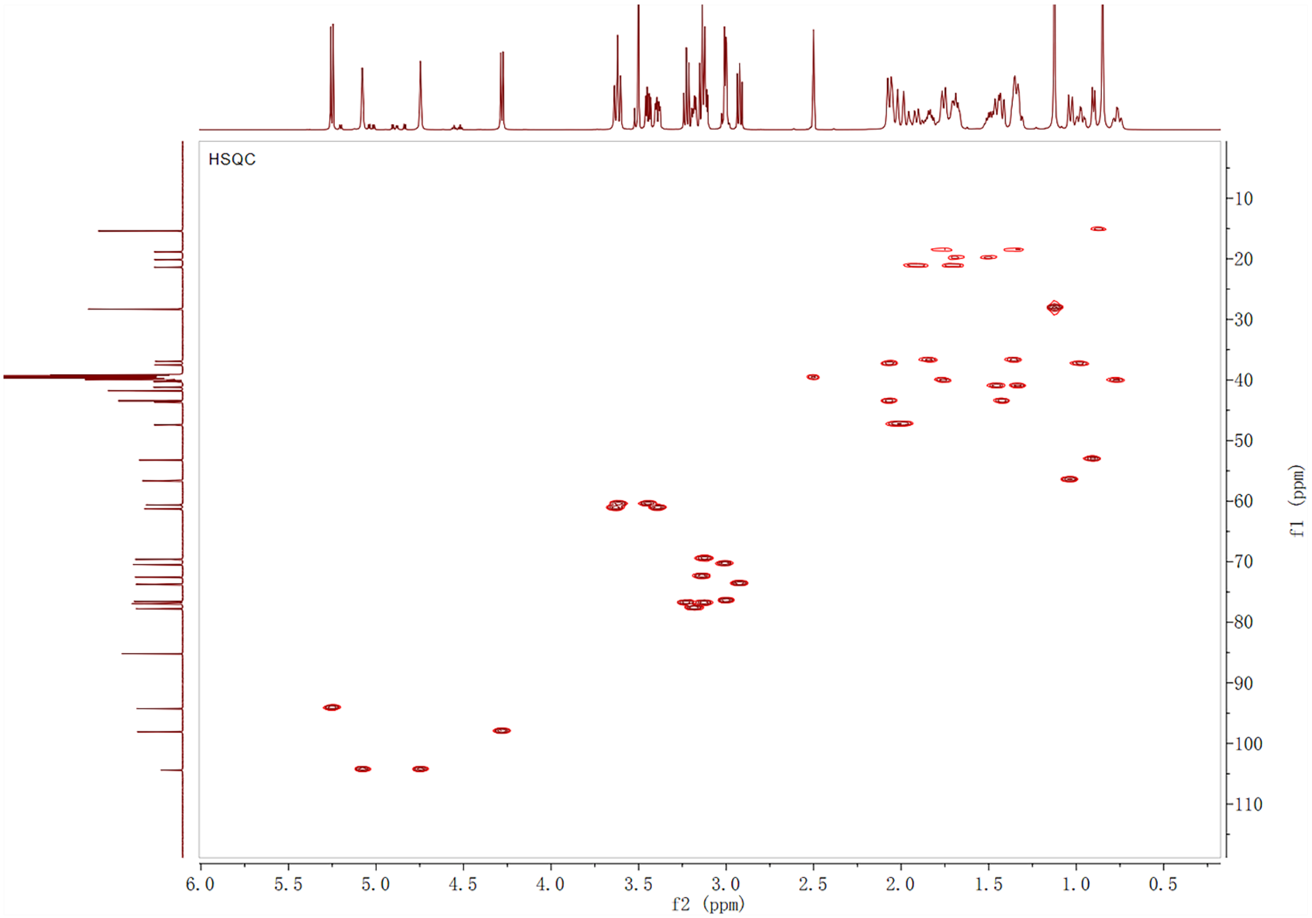

Figure S12. HSQC of the hydrolysis production from stevioside by *Cs*BGL. (δ: ppm)

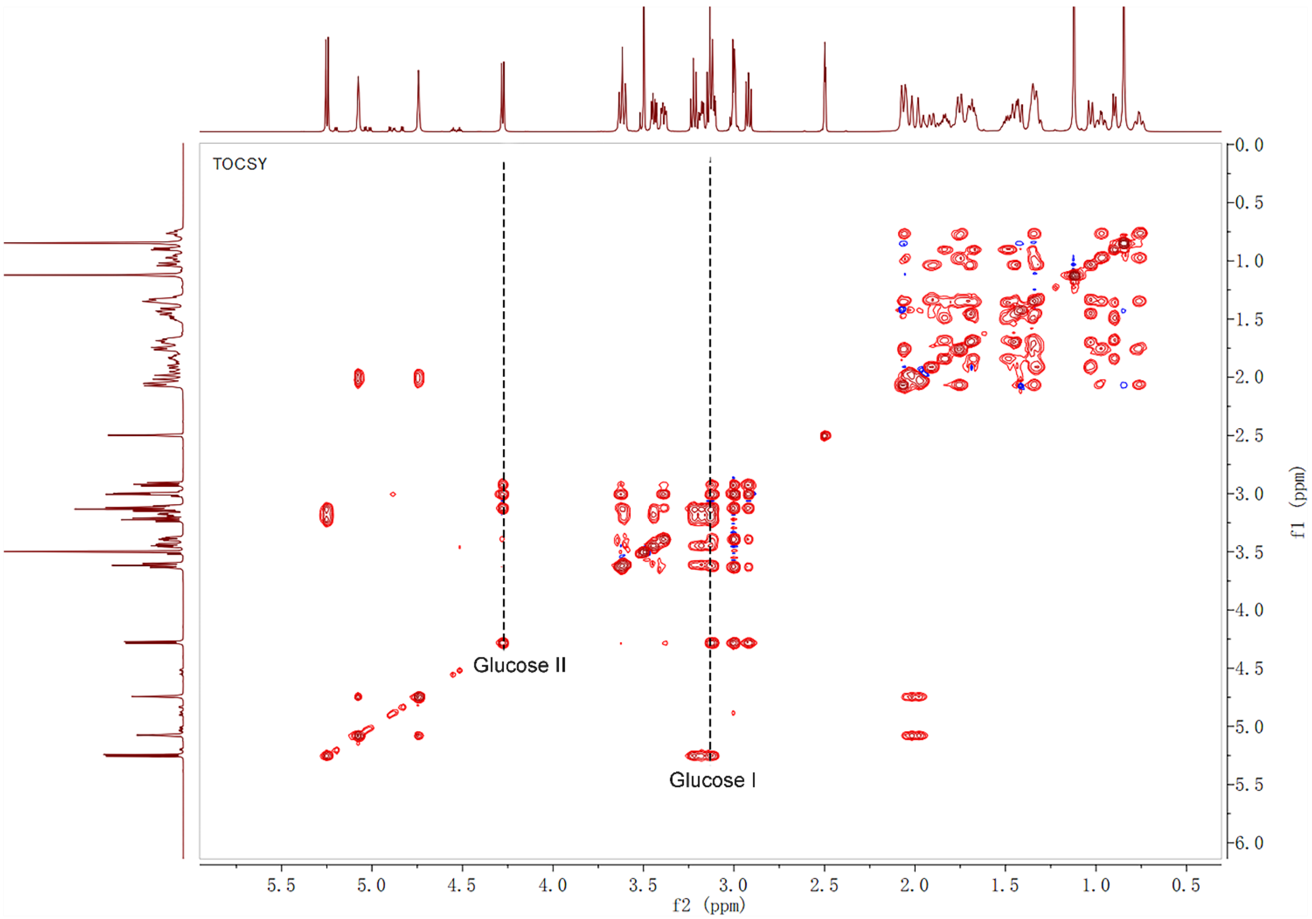

Figure S13. TOCSY of the hydrolysis production from stevioside by *Cs*BGL. (δ: ppm)

Supplementary Tables

Table S1. Chemical shifts of the hydrolysis production from stevioside by *Cs*BGL. (δ: ppm)

	**Glycon**		**Glucose I**		**Glucose II**	
	**^{1}H**	**^{13}C**	**^{1}H**	**^{13}C**	**^{1}H**	**^{13}C**
**1**	1.76, 0.76	40.27	5.25 (d, J = 8.1 Hz)	94.25	4.28 (d, J = 7.8 Hz)	98.09
**2**	1.70, 1.68	20.14	3.14	72.56	2.92	73.73
**3**	2.07, 0.97	37.52	3.24	76.90	3.12	69.60
**4**	-	43.45	3.13	76.93	3.00	76.55
**5**	1.03	56.63	3.18	77.75	3.01	70.70
**6**	1.70, 1.90	21.39	3.62, 3.45	60.62	3.63, 3.39	61.27
**7**	1.33, 1.45	41.19				
**8**	-	41.75				
**9**	0.9	53.24				
**10**	-	39.10				
**11**	1.49, 1.69	20.14				
**12**	1.36, 1.84	36.92				
**13**	-	85.19				
**14**	1.35	36.92				
**15**	2.06, 1.95	47.40				
**16**	-	153.23				
**17**	5.08, 4.74	104.39				
**18**	1.12	28.29				
**19**	-	175.92				
**20**	0.85	15.39				
